# Impact of caesarean delivery on children’s autism-like behaviours: the mediation of exclusive breastfeeding

**DOI:** 10.1186/s13006-022-00493-6

**Published:** 2022-07-15

**Authors:** Xiaoyun Qin, Peixuan Li, Ya Wu, Xiaoxu Wang, Shuangqin Yan, Yeqing Xu, Peng Zhu, Jiahu Hao, Fangbiao Tao, Kun Huang

**Affiliations:** 1 Department of Maternal, Child and Adolescent Health, School of Public Health, Anhui Medical University, Hefei, 230032 China; 2 Key Laboratory of Population Health Across Life Cycle (AHMU), MOE, Hefei, 230032 China; 3 NHC Key Laboratory of study on abnormal gametes and reproductive tract, Hefei, 230032 China; 4 Anhui Provincial Key Laboratory of Population Health and Aristogenics, Hefei, 230032 China; 5 Ma’anshan Maternal and Child Health Care Hospital, Ma’anshan, 243000 China; 6 Scientific Research Center in Preventive Medicine, School of Public Health, Anhui Medical University, Hefei, 230032 China

**Keywords:** Caesarean delivery, Autism-like behaviours, Exclusive breastfeeding, Infant, Children

## Abstract

**Background:**

The rate of autism spectrum disorder (ASD) has been rising worldwide, and therefore it is important to identify potential causes of ASD to facilitate early prevention. This study examined the role of breastfeeding and the association between caesarean delivery (CD) and children’s autism spectrum disorder.

**Methods:**

The data were from the Ma’anshan birth cohort (MABC) in China, that was set up between May 2013 and September 2014. Women within 14 gestational weeks were recruited. The delivery mode was extracted from medical notes and infant feeding was obtained from questionnaire surveys. Autism-like behaviour was assessed using the Checklist for Autism in Toddlers (CHAT-23) when children were 18 months old, and 3 years of age. At 5 years of age, autism-like behaviour was assessed using the Clancy Autism Behavior Scale behavior. Structural equation models tested the mediation effects of breastfeeding between CD and children’s autism spectrum disorder.

**Results:**

In all, 1520 (48.89%) women gave birth via CD, and 406 (13.86%) children were identified with autism-like behaviours at 18 months. Compared with women giving birth via vaginal delivery, those giving birth via CD were more likely to experience a higher proportion of delayed initiation of breastfeeding (*p* < 0.01), and delayed onset of lactogenesis (*p* < 0.01). CD was associated with a lower proportion of exclusive breastfeeding at 4 months after delivery (*p* = 0.02). Autism-like behaviour was less likely amongst infants with exclusively breastfeeding at 4 months than amongst those not exclusively breastfeeding at 4 months (*p* < 0.01). SEM indicated that women who gave birth by CD were more likely to stop exclusive breastfeeding in the first 4 months (standard estimations = − 0.04, *p* = 0.02), and those children who were not exclusively breastfed at 4 months were more likely to have autism-like behaviours (standard estimations = − 0.05, *p* < 0.01). The associations persisted at 3 years, but not at 5 years.

**Conclusions:**

Exclusive breastfeeding at 4 months of age mediated the association between caesarean delivery and children’s autism-like behaviours.

**Supplementary Information:**

The online version contains supplementary material available at 10.1186/s13006-022-00493-6.

## Background

Autism spectrum disorder (ASD) is characterized by impairment in social interaction and communication with the presence of restricted interest and repetitive behaviours [1]. The average prevalence of ASD in the United States was reported to be 1/59 among children aged 8 years in 2014 [2]. An estimated prevalence of ASD in China was reported to be 1/143 among children aged 6 to 12 years from 2014 to 2016 [3]. There has been a 20-fold increase in the diagnosis of ASD since the 1980s [2]. It may be partly due to changes in diagnostic criteria, but researchers suggested that changes in diagnostic criteria might only explain 60% of this increase [4].

ASD is associated with a large amount of life-long healthcare costs and is accompanied by various social, education, and professional adversities [5]. Recent research noted that the average annual cost per child for families amounted to €28,464.89 related to private autism spectrum disorder services, lost income and informal care [6]. A systematic analysis illustrated that years lived with disability (YLDs) for ASD in 2016 was 60,128.85 among Chinese children younger than 5 years [7]. Thus, it is crucial to identify the potential causes of ASD to facilitate for early prevention, but the exact cause is yet unclear.

Although ASD is heritable [8], recent studies have confirmed that environmental factors account for 40–50% of the causes of autism spectrum disorder [9]. Multiple studies highlighted perinatal and postnatal factors associated with the occurrence of autism [10].

The rate of caesarean delivery (CD) is increasing worldwide [11]. Much light has thus been shed on its long-term effects on children’s development, especially neuro-psychological development. Findings regarding the association between CD and children’s ASD are highly controversial. Some studies revealed that CD was associated with children’s autism spectrum disorder. Curran et al. [12] indicated that compared with vaginal delivery, children who were born by CD had a 23% increased risk of autism spectrum disorder. However, a Canadian cohort study noted that birth by CD was not associated with the increased risk of ASD among children aged 4–10 years old [13].

Breastfeeding is a dynamic, interactive and bidirectional social behavior between mothers and infants. In this process, children can not only obtain nutrients from breast milk, but also establish a lasting psychosocial connection with their mothers [14]. There are numerous benefits of breastfeeding for the children, such as providing children with key nutrients for physical and neurologic development, protecting them from infectious diseases, and decreasing their risk for allergies, asthma, and cardiovascular diseases [15, 16]. The Innocenti Declaration affirmed that all infants should receive exclusive breastfeeding from birth to 4–6 months of age [17], then WHO recommendations amended to 6 months in 2001 [18]. Studies have clarified that the mode of delivery is related to the duration of breastfeeding. Compared with women giving birth via vaginal delivery, women who gave birth by CD were more likely to experience delay in breastfeeding initiation and more likely to stop breastfeeding earlier [19–21].

Many studies found a protective effect of breastfeeding on the development of children’s autism spectrum disorder. Breast milk contains various substances, such as essential fatty acids and oxytocin, which are involved in brain development and maturation. These nutrients provide evidence for the underlying nutrition theory that breastfeeding affects the development of children’s autism spectrum disorder [22]. For instance, there is a considerable amount of long-chain polyunsaturated fatty acids (LC-PUFAs) in human milk. Studies have found that the level of PUFAs in the serum of autistic children was significantly lower than that of the control group, and that the severity of ASD could be improved after supplementing the PUFAs amongst children with autism [23, 24]. Infants’ sucking action during breastfeeding could increase maternal oxytocin levels, and oxytocin in breast milk can promote mother-infant attachment, as it is helpful for infants’ social cognition and neurodevelopment [25, 26].

In short, there is no consistent evidence between CD and offspring’s autism spectrum disorder and this study, based on a population-based birth cohort, aims to examine the role of breastfeeding in the association between CD and children’s ASD. We initiated to assess children’s autism-like behaviours at 18 months after birth under the rationale that caregivers started to note children’s behavioural development at that time [27]. Then we attempted to test whether the role of breastfeeding would persist when children were three and 5 years old.

## Methods

### Design

This was a population-based birth cohort to examine the mediation of breastfeeding in the association between CD and children’s autism spectrum disorder. This study followed the STROBE checklist for cohort studies to get clear about transparency [28] Additional file 1.

### Participants

The data presented in this paper were from the Ma’anshan birth cohort (MABC). From May 2013 to September 2014, women who had undergone their first prenatal checkup at Ma’anshan Maternal and Child Health Center were recruited. It is a tertiary center and has covered about 80 % of all pregnant women in Ma’anshan city. The criteria for women’s recruitment included: 1) over 18 years old; 2) within 14 gestational weeks; 3) willing to participate in the cohort and planned to have childbirth in the Center; 4) able to understand and answer questionnaires. Women who declined to participate were excluded from eligible population. Women with terminated pregnancies, stillbirth, twins or multiple pregnancies, forceps delivery by midwives, breech delivery by midwives and lost to follow-up for delivery methods, exclusive breastfeeding and autism-like behaviours were further excluded.

This was a ‘whole population’ study. The sample size was limited by the size of the population available in Ma’anshan. We calculated this to be adequate to detect an Odds Ratio (OR) of 1.32 with 95% confidence intervals (CI) of 1.28–1.36 [29] according to the following formula for cohort study.

$$\mathrm{n}=\frac{{\left({\mathrm{Z}}_{\upalpha}\sqrt{2\overline{\mathrm{p}\mathrm{q}}}+{\mathrm{Z}}_{\upbeta}\sqrt{{\mathrm{p}}_0{\mathrm{q}}_0+{\mathrm{p}}_1{\mathrm{q}}_1}\right)}^2}{{\left({\mathrm{p}}_1-{\mathrm{p}}_0\right)}^2}$$

Here p_0_ represented the incidence of ASD in vaginal delivery, p_1_ represented the incidence of ASD in CD, and $\overline{\mathrm{p}}$ represented the average incidence of ASD. q_0_ represented (1 − p_0_), q_1_ represented (1 − p_1_), and $\overline{\mathrm{q}}$ represented ( q_0_ + q_1_)/2). Previous studies have noted that the incidence of Autistic-like Traits was 9.6% [30], and children born by CD were more likely to experience a higher risk of ASD (OR = 1.32) after adjusting for regions, children’s sex, birth year and maternal age [29]. Therefore, p_0_ was set as 0.096, p_1_ was set as 0.127 (0.096*1.32), $\overline{\mathrm{p}}$ was set as 0.112 [(0.096 + 0.127)/2], q_0_ was set as 0.904 (1–0.096), q_1_ was set as 0.873 (1–0.128), and $\overline{\mathrm{q}}$ was set as 0.889 [(0.904 + 0.873)/2]. Additionally, α was set as 0.05, β was set as 0.10. A total of 2170 participants are required. Considering the potential rate (10 - 20%) of loss to follow-up, approximately 2713 participants would be required.

### Data collection

#### Maternal characteristics

Participated women were invited to fill in questionnaires respectively in the first, second and third trimester of pregnancy. Maternal age, education level, family income, parity and previous adverse pregnant outcomes, pregnancy complications were reported by women. Previous adverse pregnancy outcomes included spontaneous/induced abortion, premature delivery, fetal death, stillbirth, neonatal death, vaginal dystocia, hydatidiform mole, ectopic pregnancy and delivery of infants with congenital defects. Women with one or more of conditions as mentioned above were defined as having previous adverse pregnancy outcomes. Pregnancy complications included pregnancy hypertensive disorders, gestational diabetes, (overt or sub-clinical) hyperthyroidism and hypothyroidism. According to the precedent that performed in a large sample Danish cohort study [31], women with one or more of these conditions were defined as having pregnancy complications. Bodyweight and height were measured in the first trimester of pregnancy. Pre-pregnancy body mass index (BMI) was calculated by the formula BMI = weight (kg)/height^2^ (m^2^). According to the standard of China Obesity Working Group [32], pre-pregnancy BMI was classified as underweight (BMI < 18.5 kg/m^2^), normal (18.5 kg/m^2^ ≤ BMI < 24.0 kg/m^2^), overweight (24.0 kg/m^2^ ≤ BMI < 28 kg/m^2^) and obesity (BMI ≥ 28.0 kg/m^2^).

The delivery mode and complications during childbirth were extracted from medical notes. Delivery mode was classified into normal vaginal delivery and CD. CD covered elective or emergency CD, and elective CD included elective CD with or without medical indications.

Complications during childbirth included fetal distress, placental abruption, placenta previa and premature rupture of membranes. Women with one or more of these conditions were defined as having complications during childbirth.

#### Children’s characteristics

Newborn’s gender, birth weight, gestational age and Apgar scores were obtained from the medical notes. Preterm birth was defined as infants born before 37 gestational weeks. Birthweight less than 2500 g was defined as low birthweight, and more than 4000 g was defined as macrosomia.

Information on infants’ breastfeeding was obtained from questionnaire surveys at 42 days and 6 months after childbirth. Breastfeeding parameters included the initiation of breastfeeding, the onset of lactogenesis and exclusive breastfeeding at 4 months and 6 months after delivery. Based on WHO guidelines, exclusive breastfeeding was defined as infants were provided no other liquid or solid foods (including water) except for breast milk [33].

Autism-like behaviour was assessed using the Checklist for Autism in Toddlers (CHAT-23) when children were 18 months old [34] (Additional file 2: Appendix 2). CHAT-23 is widely used to distinguish between children with and without autism because its high sensitivity and specificity [34]. It contained Section A (parental questionnaire) and Section B (observational checklist). Section A was a self-administered questionnaire by main caregivers; thus, it would be the first choice for screening as it is simple and easy to conduct in a large-sample field investigation [34]. Section A was therefore used to assess children’s autism-like behaviours in the current study. It contained 23 items (7 core items). Two criteria were reported for autism-like behaviours in section A, including the total score ≥ 6 (sensitivity was 0.839; specificity was 0.848; positive predictive value (PPV) was 0.793) and the score of core items ≥2 (sensitivity was 0.931; specificity was 0.768; PPV was 0.736) [34]. In this study, children with autism-like behaviours were defined as meeting either of the criteria. The Cronbach α coefficient was 0.869, and the split-half reliability coefficient was 0.865 in the current study.

Children’s autism-like behaviours were re-assessed by using CHAT-23 at 3 years old. When children were at 5 years old, the modified Chinese version of the Clancy Autism Behavior Scale was adopted to assess children’s autism-like behaviours. This scale was widely used in mainland China, and the sensitivity, specificity, PPV was 58, 84, 65%, respectively [35].

#### Quality-control

In MABC, we have made a series of strategies to reduce the loss of follow-up: 1) During the process of informed consents, all participants were informed in detail of aim of the study, frequencies and contents of follow-ups for both mothers and infants. By that, women clearly knew what to do, how often to do and what the potential rights and risks would bring, and decided whether or not to participate. 2) During the follow-up period, we used an electronic participants management system, in which the due date of each participant’s follow-up would automatically present 1week in advance. Our team members would call the participants and make the appointment for the coming follow-ups. If they did not come on schedule, telephone calls would be made at least 1 week, 2 weeks and 1 month after the due date until they finally came. 3) In addition, we have taken some measures to encourage participants for follow-ups. For instance, “green channels” (quick arrangements without waiting) were set for cohort women for their prenatal checkups and hospitalized deliveries. Free assessments of physical and psychological development would be provided to children in the cohorts. We also established online communication platforms to communicate with participants and provide consultations and supports if they had encountered any questions or issues during pregnancy and infants’ caregiving.

### Data analysis

Data were analyzed by using IBM SPSS Statistics 25 and Amos 23.0 software. *χ*^2^ tests/t tests were used to compare the maternal, children’s characteristics and breastfeeding parameters between different delivery modes. These tests were also adopted to identify differences in the prevalence of autism-like behaviours between different delivery modes and different exclusive breastfeeding statuses.

The structural equation model was used to assess the internal structure of studying variables (the mediation effects of breastfeeding between CD and children’s ASD) [36]. Corresponding variables with *p* values over 0.1 in correlation analysis were included in the structural equation models. The relative strength of each hypothesized relationship was indicated by its standardized coefficients. These values were in the range of − 1.0 to + 1.0. The adequacy of the fit of the hypothesized model to the observed data was reported using the model likelihood ratio goodness-of-fit chi-square statistic. As this statistic tended to over-estimate departures from a good model fit with large sample sizes, the following more informative statistics were also reported: goodness-of-fit chi-square (*χ*^*2*^); degrees of freedom (*df*); *χ*^*2*^ */ df* should be < 5 and > 2; Root Mean Square Error of Approximation (RMSEA), the value of which should be ≤0.08; Normal Fit Index (NFI), the value of which should be ≥0.90; Comparative Fit Index (CFI) and Incremental fit index (IFI), the value of which should also be ≥0.90 [37–39].

Considering that assisted vaginal births may have a potential effect on children’s development [40], we conducted sensitivity analyses including the 14 children born via assisted deliveries and re-ran all the analyses when children were 18 months old.

## Results

### Number of participants

**Fig. 1:**
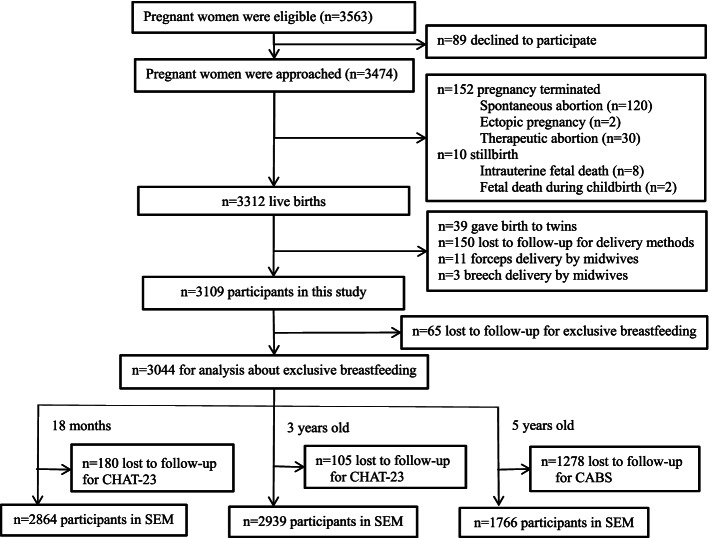
Flowchart of participant recruitment. SEM: Structural equation model. CHAT-23: the Checklist for Autism in Toddlers. CABS: the modified Chinese version of the Clancy Autism Behavior Scale

The numbers in the cohort eligible were 3563, and 89 declined to participate during informed consents. A total of 3474 pregnant women were recruited in the cohort. After exclusion of terminated pregnancies (152), stillbirth (10), twins or multiple pregnancies (39), forceps delivery by midwives (11), breech delivery by midwives (3), lost to follow-up for delivery methods (150), lost to follow-up for exclusive breastfeeding (65), lost to follow-up for autism-like behaviours (180), a total of 2864 mother-child pairs were finally included in structural equation model when children were at 18 months after birth (Fig. 1**)**. In accordance with the same procedure of participants recruitment at 18 months, totally 2939 and 1766 mother-child pairs were included for analysis respectively at 3 and 5 years old (Fig. 1).

### Basic characteristics of participants

When children were 18 months old, among the participated women, the mean age was 26.61 years and the mean education years were 13.37. Most were primiparous (90.16%) and did not have pregnancy complications (93.63%) or complications during childbirth (93.63%).

Among the participants, 48.89% (1520/3109) women gave childbirth by caesarean delivery. The rate of exclusive breastfeeding at 4 months and 6 months was 44.97% (1288/2864) and 10.17% (305/2998), respectively. There were 13.86% (406/2929) of children with autism-like behaviours.

Among 2939 3-year-old children who were re-assessed by CHAT-23, 409 (13.92%) children were observed to have autism-like behaviours. All the 406 children who were screened as to be with autism-like behaviours at 18 months were among these 409 children. In 1766 5-year-old children who were assessed by the Clancy Autism Behavior Scale, 86 (4.87%) of them were found to have autism-like behaviours. There were 237 children among 406 children with autism-like behaviours at 18 months, and 15 (6.33%) of them were found to have autism-like behaviours.

### Basic maternal and children’s characteristics between different delivery modes

As shown in Table 1, compared with those who gave birth by vaginal deliveries, women who gave birth by CD were older, multiparous, and were more likely to stay higher pre-pregnancy body mass index. These women that gave birth via CD were more likely to experience a higher rate of previous adverse pregnancy outcomes and pregnancy complications. Children born by CD were more likely to keep birthweight over 4000 g than those born of vaginal delivery.

**Table 1:** Maternal and children’s characteristics between different delivery modes (*n* = 3109)

Maternal and children’s characteristics	Total populations	Delivery modes		Missing (n/%)	***df***	***t/χ***^***2***^ values	***p*** values
		Vaginal delivery	Caesarean delivery				
**Maternal characteristics**							
Age (years) (Mean ± SD)	26.61 ± 3.66	26.17 ± 3.26	27.09 ± 3.94	0/0.0	2950	−7.11	< 0.01
Maternal education level (years) (Mean ± SD)	13.37 ± 3.16	13.44 ± 3.18	13.3 ± 3.14	20/0.6	3087	1.40	0.16
Monthly income per capita (Yuan) (n/%)				0/0.0	2	2.61	0.27
≤ 2499	829/26.66	439/27.62	390/25.66				
2500 ~ 3999	1326/42.65	681/42.86	645/42.43				
≥ 4000	954/30.69	469/29.52	485/31.91				
Pre-pregnancy BMI (kg/m^2^) (n/%)				47/1.51	3	60.52	< 0.01
Underweight	702/22.58	420/26.77	282/18.89				
Normal	2028/65.23	1033/65.84	995/66.64				
Overweight	274/8.81	103/6.56	171/11.45				
Obesity	58/1.87	13/0.83	45/3.01				
Parity (n/%)				0/0.0	1	8.63	< 0.01
Primiparous	2803/90.16	1457/91.69	1346/88.55				
Multiparous	306/9.84	132/8.31	174/11.45				
Previous adverse pregnancy outcomes (n/%)				0/0.0	1	19.94	< 0.01
Had	1292/41.56	599/37.70	693/45.59				
Did not have	1817/58.44	990/62.30	827/54.41				
Pregnancy complications (n/%)				0/0.0	1	13.71	< 0.01
Had	198/6.37	76/4.78	122/8.03				
Did not have	2911/93.63	1513/95.22	1398/91.97				
Complications during childbirth (n/%)				13/0.42	1	3.07	0.08
Had	826/26.67	400/25.3	426/28.1				
Did not have	2270/73.01	1180/74.7	1090/71.9				
**Children’s characteristics**							
Gender				0/0.00	1	2.23	0.14
Boys	1593/51.24	835/52.55	758/49.87				
Girls	1516/48.76	754/47.45	762/50.13				
Birth weight (g) (n/%)				0/0.00	2	44.66	< 0.01
< 2500	75/2.41	39/2.45	36/2.37				
2500 ~ 3999	2823/90.80	1489/93.71	1334/87.76				
≥ 4000	211/6.79	61/3.84	150/9.87				
Gestational weeks (n/%)				0/0.0	1	3.72	0.05
< 37	126/4.05	75/4.72	51/3.36				
≥ 37	2983/95.95	1514/95.28	1469/96.64				
1-minute Apgar scores (Mean ± SD)	9.94 ± 0.52	9.94 ± 0.45	9.93 ± 0.59	42/1.35	3065	0.94	0.35
5-minute Apgar scores (Mean ± SD)	9.98 ± 0.20	9.98 ± 0.23	9.99 ± 0.18	43/1.38	2950	−1.39	0.17

### Breastfeeding parameters between different delivery modes

Compared with women giving birth by vaginal delivery, those who gave birth by CD were more likely to undergo a higher proportion of delayed initiation of breastfeeding, and women who gave birth via CD were less likely to initiate breastfeeding within 30 minutes after childbirth than that of those with vaginal delivery. Delayed onset of lactogenesis was observed in women giving birth via caesarean delivery. Compared with those who gave birth by vaginal deliveries, women who gave birth via CD were less likely to lactate breast milk in the delivery room and within the day of childbirth, but were more likely to start lactation at 3 days after delivery. CD was associated with a lower proportion of exclusive breastfeeding at 4 months after delivery. There was no significant difference between exclusive breastfeeding at 6 months and different modes of delivery Table 2.

**Table 2:** Breastfeeding parameters among women with different delivery modes [(n, %)]

Breastfeeding parameters	n	Delivery modes		*df*	*χ*^*2*^ value	*p* value
		Vaginal delivery	Caesarean delivery			
Initiation of breastfeeding					170.25^a^	< 0.01
Within 30 minutes after delivery	1010	649/40.97	361/23.94	1		
Within 1 hour after delivery	328	177/11.17	151/10.01			
Within 2 hours after delivery	186	133/8.40	53/3.51			
Within 3 hours after delivery	103	63/3.98	40/2.65			
Within the day of delivery	704	290/18.31	414/27.45			
Within longer time	761	272/17.17	489/32.43			
Onset of lactogenesis				1	81.67^a^	< 0.01
In the delivery room	330	241/15.21	89/5.89			
Within the day of delivery	685	378/23.86	307/20.33			
Within the first day after delivery	385	209/13.19	176/11.66			
Within the second day after delivery	682	327/20.64	355/23.51			
Within the third day after delivery	737	301/19.00	436/28.87			
Within longer time	275	128/8.08	147/9.74			
Exclusive breastfeeding at 4 months				1	5.90	0.02
Yes	1348	721/47.12	627/42.71			
No	1650	809/52.88	841/57.29			
Exclusive breastfeeding at 6 months				1	0.20	0.67
Yes	305	152/9.93	153/10.42			
No	2693	1378/90.07	1315/89.58			

^a^linear-by-linear association

### Children’s autism-like behaviours in different delivery modes and exclusive breastfeeding durations at 18 months

As shown in Table 3, there was no significant difference between autism-like behaviours and children’s delivery modes. Autism-like behaviour was less likely amongst infants with exclusively breastfeeding at 4 months than amongst those not exclusively breastfeeding at 4 months. There was no statistically significant difference between autism-like behaviour and exclusively breastfeeding at 6 months.

**Table 3:** Children’s autism-like behaviours in different delivery modes and exclusive breastfeeding durations at 18 months (n/%)

Groups	Delivery modes		Exclusive breastfeeding at 4 months		Exclusive breastfeeding at 6 months	
	Vaginal delivery	Caesarean delivery	Yes	No	Yes	No
Children with autism-like behaviours	207/13.85	199/13.88	153/11.88^**^	243/15.42	26/9.15	370/14.34
Children without autism-like behaviours	1288/86.15	1235/86.12	1135/88.12	1333/84.58	258/90.85	2210/85.66

^**^p < 0.01

### Mediation effect of exclusive breastfeeding at 4 months between CD and children’s autism-like behaviours

**Fig. 2:**
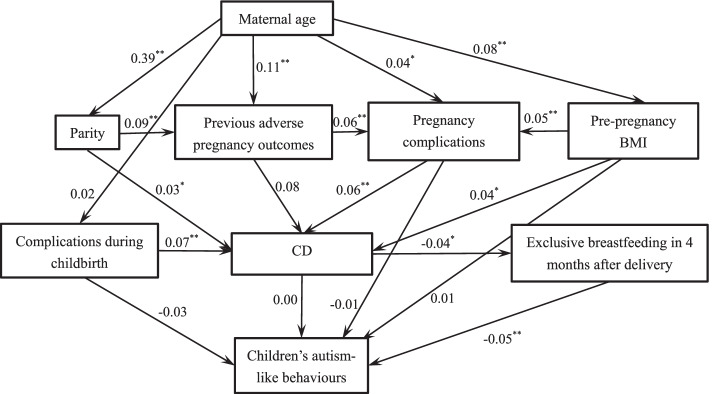
Structural equation model showing the mediation effect of exclusive breastfeeding within 4 months after delivery on the association between CD and children’s autism-like behaviours at 18 months. CD: Caesarean delivery. *: *p* < 0.05. **: *p* < 0.01. The coefficients on the arrow are standardized estimations

The final hypothesized structural equation model, relating maternal age, parity, pre-pregnancy BMI, previous adverse pregnancy outcomes, pregnancy complications, complications during childbirth, CD, exclusive breastfeeding at 4 months to autism-like behaviours at 18 months was shown in Fig. 2. The model fit indices values were: *χ*^*2*^ = 74.028, *df* = 17; *χ*^*2*^***/****df* = 4.355; *p* < 0.001; RMSEA = 0.033; CFI = 0.927, IFI = 0.929, NFI = 0.910. The model fit was basically good.

Strong associations were observed between maternal age, parity, previous adverse pregnancy outcomes, pregnancy complications and caesarean delivery. Women who were older were more likely to be multiparous, multiparous women would be more likely to have previous adverse pregnancy outcomes, previous adverse pregnancy outcomes would be related to more pregnancy complications, and women with more pregnancy complications would be more possible to give birth via caesarean delivery. And then, women who gave birth via CD were more likely to stop exclusive breastfeeding at 4 months, and finally those children who didn’t receive exclusively breastfeeding at 4 months after delivery were more likely to have autism-like behaviours at 18 months. There was no direct relationship between CD and children’s autism-like behaviours at 18 months. A mediation effect of exclusive breastfeeding at 4 months between CD and toddlers’ autism-like behaviours was observed Table 4.

**Table 4:** Estimations of structural equation model on mediation effect of exclusive breastfeeding at 4 months between CD and children’s autism-like behaviours at 18 months (*n* = 2864)

Variables	Unstandardized estimations	Standardized estimations	***p*** values
maternal age -- parity	0.03	0.39	< 0.01
parity-- previous adverse pregnancy outcome	0.15	0.09	< 0.01
maternal age -- previous adverse pregnancy outcome	0.02	0.11	< 0.01
maternal age -- pre-pregnancy BMI	0.03	0.08	< 0.01
maternal age -- pregnant complications	0.00	0.04	0.03
pre-pregnancy BMI-- pregnant complications	0.00	0.05	< 0.01
previous adverse pregnancy outcome -- pregnant complications	0.03	0.06	< 0.01
maternal age --complications during childbirth	0.00	0.02	0.29
pregnant complications -- caesarean delivery	0.13	0.06	< 0.01
parity--caesarean delivery	0.07	0.03	0.02
pre-pregnancy BMI -- mode of delivery	0.03	0.04	0.02
complications during childbirth-- caesarean delivery	0.03	0.07	< 0.01
previous adverse pregnancy outcome --caesarean delivery	0.08	0.08	0.14
caesarean delivery -- exclusive breastfeeding at 4 months	− 0.03	− 0.04	0.02
caesarean delivery -- children’s autism-like behaviours	0.00	0.00	0.91
exclusive breastfeeding at 4 months -- children’s autism-like behaviours	− 0.04	− 0.05	< 0.01
pre-pregnancy BMI -- children’s autism-like behaviours	0.01	0.01	0.36
complications during childbirth -- children’s autism-like behaviours	−0.02	− 0.03	0.84
pregnant complications -- children’s autism-like behaviours	−0.01	−0.01	0.59

Sensitivity analyses revealed no substantial change from the main findings (data not shown).

**Fig. 3:**
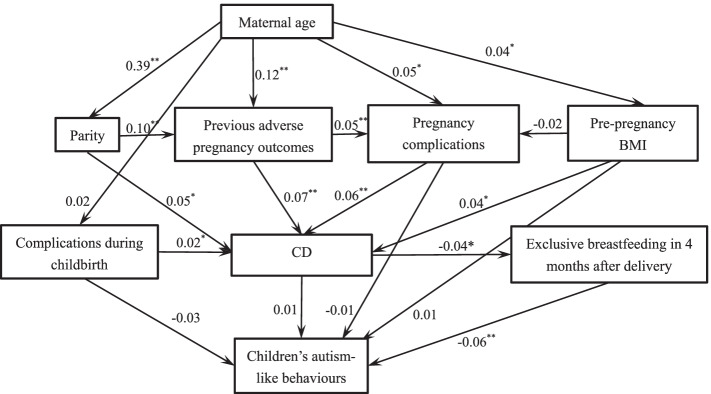
Structural equation model showing the mediation effect of exclusive breastfeeding within 4 months after delivery on the association between CD and children’s autism-like behaviours at 3 years. CD: Caesarean delivery. *: *p* < 0.05. **: *p* < 0.01. The coefficients on the arrow are standardized estimations

The structural equation model in 3-year-old children was re-conducted and shown in Fig. 3. The model fit indices: *χ*^*2*^ = 77.736, *df* = 17, *χ*^*2*^/ *df* = 4.573, *p* < 0.001, RMSEA = 0.036, CFI = 0.907, IFI = 0.910, NFI = 0.887. The same mediation effect of exclusive breastfeeding at 4 months between CD and 3-year-old children’s autism-like behaviours was observed (Table 5).

**Table 5:** Estimations of structural equation model on mediation effect of exclusive breastfeeding at 4 months between CD and children’s autism-like behaviours at 3 and 5 years

Variables	3-year-old children (***n*** = 2939)			5-year-old children (***n*** = 1766)		
	Unstandardized estimations	Standardized estimations	***p*** values	Unstandardized estimations	Standardized estimations	***p*** values
maternal age -- parity	0.03	0.39	< 0.01	0.03	0.38	< 0.01
parity-- previous adverse pregnancy outcome	0.17	0.10	< 0.01	0.14	0.08	< 0.01
maternal age -- previous adverse pregnancy outcome	0.02	0.12	< 0.01	0.02	0.16	< 0.01
maternal age -- pre-pregnancy BMI	0.01	0.04	0.02	0.02	0.15	< 0.01
maternal age -- pregnant complications	0.00	0.05	0.01	0.00	0.07	< 0.01
pre-pregnancy BMI-- pregnant complications	−0.01	−0.02	0.43	0.01	0.02	0.52
previous adverse pregnancy outcome -- pregnant complications	0.03	0.05	< 0.01	0.02	0.04	0.10
maternal age --complications during childbirth	0.00	0.02	0.39	0.00	0.03	0.20
pregnant complications -- caesarean delivery	0.13	0.06	< 0.01	0.14	0.07	< 0.01
parity--caesarean delivery	0.08	0.05	0.01	0.02	0.01	0.64
pre-pregnancy BMI -- caesarean delivery	0.03	0.04	0.04	0.11	0.13	< 0.01
complications during childbirth-- caesarean delivery	0.03	0.02	0.02	0.00	0.00	1.00
previous adverse pregnancy outcome --caesarean delivery	0.07	0.07	< 0.01	0.08	0.08	< 0.01
caesarean delivery -- exclusive breastfeeding at 4 months	−0.04	−0.04	0.04	−0.03	−0.03	0.29
caesarean delivery -- children’s autism-like behaviours	0.00	0.01	0.79	0.00	0.01	0.72
exclusive breastfeeding at 4 months -- children’s autism-like behaviours	−0.04	− 0.06	< 0.01	0.00	− 0.01	0.72
pre-pregnancy BMI -- children’s autism-like behaviours	0.01	0.01	0.55	0.00	0.00	0.94
complications during childbirth -- children’s autism-like behaviours	−0.02	−0.03	0.19	0.02	0.04	0.12
pregnant complications -- children’s autism-like behaviours	−0.01	−0.01	0.80	−0.04	− 0.04	0.10

**Fig. 4:**
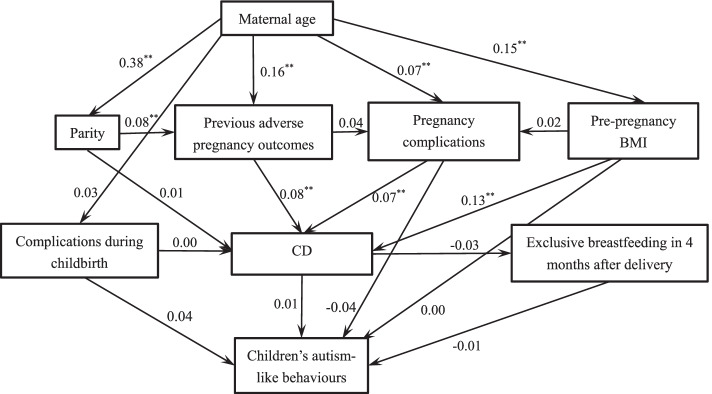
Structural equation model showing the mediation effect of exclusive breastfeeding within 4 months after delivery on the association between CD and children’s autism-like behaviours at 5 years. CD: Caesarean delivery. *: *p* < 0.05. **: *p* < 0.01. The coefficients on the arrow are standardized estimations

Figure 4 indicated the final structural equation model for 5-year-old children. The model fit indices values were: *χ*^*2*^ = 41.646, *df* = 17, *χ*^*2*^/*df* = 2.450, *p* < 0.001, RMSEA = 0.029, CFI = 0.946, IFI = 0.949, NFI = 0.916. The mediation effect of exclusive breastfeeding at 4 months between CD and autism-like behaviours did not persist (Table 5).

## Discussion

In this study, a lower rate of exclusive breastfeeding was identified in women with CD, and exclusive breastfeeding at 4 months after delivery was found to be related to a low prevalence of children’s autism-like behaviours. Exclusive breastfeeding at 4 months after childbirth may mediate the association between CD and children’s autism-like behaviours within 3 years old.

The CD rate in our study was 48.89%, which was much higher than those of other countries in the world, such as Japan (18.6%) and the United States (32.0%) [41]. It was higher than the rate in China in 2011–2012 (46.2%) [41] and in 2015–2016 (38.9%) [42], but was lower than the CD rate in 2016–2019 in Jiangsu, China (49.8%) [43]. Our previous qualitative study has revealed that maternal fear of pain, worry about mothers’ and infants’ safety, dissatisfaction with doctors’ competencies and physicians’ low confidence in vaginal delivery, and absence of strong midwifery care together contributed to the final determination of caesarean delivery [44]. Findings from another research group also supported the above-mentioned reasons [45]. With the stubbornly high CD rate in China, there are a large number of reports on the impact of CD on maternal and offspring’s health, including children’s behavioural development.

### The relationship between CD and children’s autism-like behaviours

Previous studies have reported inconsistent findings on the relationship between caesarean delivery and children’s autism disorders as shown in Table 6. Findings from three studies were consistent with our study that no significant effect of CD was observed on children’s autism-like behaviours. They had a longer follow-up than the current study, and the eldest children were 15 years old [13, 46, 47]. In contrast, a Western Australia study investigated the offspring at the age of 5–25 years and found that CD was consistently associated with a modestly increased risk of autism spectrum disorder [29]. A recent Malaysian study revealed that 7-year-old children born with CD had a higher risk of autism than those born via normal delivery [48]. The contradictory findings may be due to the differences in sample size, the introduction of adjustments and assessment instruments for autism spectrum disorder. Due to the difficulties in conducting randomized controlled trials to examine the association between CD and children’s ASD, prospective cohort studies with large samples are urgently in need.

**Table 6:** The characteristics of relevant literatures focusing on the association between CD and ASD

Authors	Publication year	Research area	Sample size	Offspring’s age	Instrument for ASD assessment	Association	Main findings
Andoy Galvan JA et al. [48]	2020	Malaysia	929	9y	DSM or ICD10	Positive	The odds of autism were higher among those born via CD compared to those born via normal deliveries.
Yip et al. [29]	2017	Western Australia	345,181	5-25y	ICD-8, ICD-9, ICD-10, and DSM-IV	Positive	CD was consistently associated with a modest increased risk of ASD.
Burstyn et al. [13]	2010	Canada	220,028	4-10y	ICD-9 codes 299.0 and 299.8	Negative	There was no association between mode of delivery and ASD.
Curran et al. [46]	2016	Britain	13,141	7y	Parent report	Negative	No association between mode of delivery and ASD was observed.
Curran et al. [47]	2015	Sweden	26,822	7-15y	ICD-9 code 299 and ICD-10 code F84	Negative	Children born by CS were found to be approximately 20% more likely diagnosed as having ASD. However, the association did not persist when using sibling controls, implying that this association is due to familial confounding by genetic and/or environmental factors.

*CD:* Caesarean delivery

*ICD:* International Classification of Diseases

*DSM:* Diagnostic and Statistical Manual of Mental Disorders

### The relationship between CD and breastfeeding parameters

Our findings revealed that delayed initiation of breastfeeding, delayed onset of lactogenesis and the short duration of exclusive breastfeeding related to CD, as could be supported by many other studies [19, 20]. Studies have also noted that women who delivered by CD may have less intention to breastfeed or did not initiate breastfeeding compared with those who delivered vaginally (7.4 and 4.3% respectively) [19]. CD might cause lots of postpartum pain and may delay the initiation of breastfeeding [20]. On the other hand, it is known that infants’ sucking could stimulate women’s secretion of prolactin and would then stimulate breast-milk production. Delayed skin contact between mothers and infants occurs more frequently in CD than between mother-infant pairs in vaginal delivery, and this would also prolong the initiation of breastfeeding after CD [21]. Delayed onset of lactation in CD may play a regulatory role in leading to short breastfeeding duration [19].

### Mediation effect of exclusive breastfeeding in the association between CD and children’s autism-like behaviours

Rates of ASD varied widely among studies with the usage of different assessments. Center for Disease Control and Prevention announced that the rate of ASD was 1/59 in the US by using the Diagnostic and Statistical Manual of Mental Disorders, Fourth Edition Text Revision (DSM-IV-TR) [2]. Zhou H et al. [3], by using a modified Chinese version of the Autism Spectrum Rating Scale (MC-ASRS), have reported that the prevalence of ASD is about 6.9/1000 in Chinese children. In this study, with a screening questionnaire, CHAT-23, the prevalence of autism-like behaviours in children aged 18 months was found to be 13.86%. The specificity of the screening scale might be lower than that of DSM-IV [49]. In the current study, we did not indicate a direct effect of CD on children’s autism-like behaviours, but a mediation effect of exclusive breastfeeding at 4 months after delivery was identified. Many studies found shorter breastfeeding duration in children with ASD versus children without ASD, which was consistent with our results, suggesting a possible protective effect of breastfeeding. Al-Farsi et al. [50] revealed that late initiation of breastfeeding and the absence of colostrum were related to children’s later occurrence of ASD in a dose-response manner. A sibling-controlled study found that the breastfeeding rate in children with ASD was significantly lower than those of controls [22]. Recent studies also observed a significant association between the short duration of breastfeeding and the presence of children’s ASD [51].

Detection of behavioural features at 18 months represents an age by which many caretakers begin to note concerns regarding their children’s development. This may provide an early time window to identify children’s abnormal behavioural development, as well as the subsequent opportunity of early intervention for these children. It is the original motivation to perform this study. In spite of this, we have performed repetitive analyses by using children’s later follow-ups when they were three and 5 years old. The same findings were observed when children were 3 years old, while the mediation effect did not persist when children reached 5 years. One may speculate that compared with children at 18 months and 3 years old, less 5-year-old children were assessed, with a different instrument. The underlying reason that the mediation effect of breastfeeding does not persist beyond 3 years was unclear. This will be explored in another paper towards the interesting question as to why autistic traits disappeared in some children, but not others.

### Strengths and limitations

This study has some strengths. First of all, previous studies only considered the isolated impact of CD or breastfeeding on children’s autism spectrum disorder. This study, for the first time, jointly investigated CD, exclusive breastfeeding and children’s autism-like behaviours. Meanwhile, most potential confounders and their possible interactions were considered in the structural equation models. Secondly, this study was a prospective cohort study. Compared with retrospective or cross-sectional study design, data of exposure, outcome and other covariates were more precise, and the conclusion was thus more credible. In the third, there are few studies looking into children’s autism-like behaviours at the age of 18 months, and this may provide an early time window to identify children’s abnormal behavioural development, as well as an early intervention for these children.

This study also has some limitations. Firstly, the type of CD could not be classified into elective or emergency CD in the structural equation models. In some cases, the reasons to conduct an emergency CD, such as fetal distress, may be the exact cause, other than CD, for children’s abnormal development in later life [52], so confounding by indication could not be fully ruled out in this study. In the second, by using a screening scale, the prevalence of real autism spectrum disorders could not be available in the current study. Although CHAT-23 is proved to be suitable for large-sample population-based studies, with a PPV of 0.793 (total score ≥ 6) [34], it was inevitable that there could be some false positive cases, which would bias our findings. In the third, it was important to use both section A and B during screening for autism among children. Due to insufficient professional evaluators, Section B was not used in our study, but it is reported that either section A or B could identify children with autism-like behaviours [34]. Fourthly, studies have demonstrated medicines in labor could affect infants’ feeding, such as exogenous oxytocin and opioids (intrapartum fentanyl). Either of the two kinds of medicines is associated with less optimal breastfeeding outcomes [53, 54]. Intrapartum medication should be included as an important element of infant feeding. However, we did not have the full data on medication during labor when abstracting the information from medical notes. Fifthly, this study was nested in Ma’anshan birth cohort, and the original design was to investigate the association between multiple environmental exposures in early life and children’s development. The calculation of sample size was retrospective. However, the number of participants sample size in this study was larger than the required sample size at 18 months and 3 years. Then, while structural equation models allow one to assess many effects, they also require that the relations between all variables are un-confounded [55]. The confounding assumptions described in our study were needed not just for a single exposure, mediator or outcome, but for every set of variables in the model. It could be useful for hypothesis generation but still needed to be interpreted cautiously. Furthermore, the rate of exclusive breastfeeding at 6 months was 10.17%, as would affect statistical interpretations in the association between CD and children’s autism-like behaviours. Finally, due to the high CD rate in this study, the conclusion needs to be further verified in other countries and regions with low CD rate.

## Conclusions

Caesarean delivery relates to a delayed initiation and short duration of breastfeeding. Exclusive breastfeeding at 4 months after childbirth may mediate the association between CD and children’s autism-like behaviours within 3 years. The findings, if causal, will provide an integrated perspective for the prevention of children’s autism-like behaviours.

## Supplementary Information

**Additional file 1.**

**Additional file 2.**

## Data Availability

The dataset from the current study is available from the corresponding author on reasonable request.

## Abbreviations

ASD: Autism spectrum disorder
BMI: Body mass index
CD: caesarean delivery
CFI: Comparative Fit Index
CHAT-23: Checklist for Autism in Toddlers
DSM-IV-TR: Diagnostic and Statistical Manual of Mental Disorders, Fourth Edition Text Revision
IFI: Incremental fit index
LC-PUFAs: Long-chain polyunsaturated fatty acids
MABC: Ma’anshan birth cohort
MC-ASRS: Chinese version of the Autism Spectrum Rating Scale
NFI: Normal Fit Index
PPV: Positive predictive value
RMSEA: Root Mean Square Error of Approximation
SEM: Structural equation model
UNICEF: United Nations Children’s Fund
