# Supplementary material for: Impact of caesarean delivery on children’s autism-like behaviours: the mediation of exclusive breastfeeding
Source: Int Breastfeed J. 2022 Jul 15;17:53. doi: 10.1186/s13006-022-00493-6 (PMC9284736; doi:10.1186/s13006-022-00493-6)
Supplement: Supplementary file 2 — Additional file 2. [file 13006_2022_493_MOESM2_ESM.docx]

**Appendix 2.**

**English Version of the CHAT-23**

| Section A: Self-Administered Questionnaire | | | | |
| --- | --- | --- | --- | --- |
| Please fill out the following about how your child usually is. This questionnaire should be administered by the chief caregiver of the child. Please fill the corresponding circle black completely. | | | | |
|  | Never | Rarely | Sometimes | Often |
| 1. Does your child enjoy being swung, bounced on your knee, etc? | ○ | ○ | ○ | ○ |
| 2. Does your child take an interest in other children? | ○ | ○ | ○ | ○ |
| 3. Does your child like climbing on things, such as up stairs? | ○ | ○ | ○ | ○ |
| 4. Does your child enjoy playing peck-a-boo/hide-and-seek? | ○ | ○ | ○ | ○ |
| 5. Does your child ever pretend, for example, to talk on the phone or take care of dolls, or pretend other things? | ○ | ○ | ○ | ○ |
| 6. Does your child ever use his/her index finger to point, to ask for something? | ○ | ○ | ○ | ○ |
| 7. Does your child ever use his/her index finger to point, to indicate interest in something? | ○ | ○ | ○ | ○ |
| 8. Can your child play properly with small toys (eg, cars or bricks) without just mouthing, fiddling, or dropping them? | ○ | ○ | ○ | ○ |
| 9. Does your child ever bring objects over to you (parent) to show you something? | ○ | ○ | ○ | ○ |
| 10. Does your child look you in the eye for more than a second or two? | ○ | ○ | ○ | ○ |
| 11. Does your child ever seem oversensitive to noise? (eg, plugging ears) | ○ | ○ | ○ | ○ |
| 12. Does your child smile in response to your face or your smile? | ○ | ○ | ○ | ○ |
| 13. Does your child imitate you? (eg, you make a face; will your child imitate it?) | ○ | ○ | ○ | ○ |
| 14. Does your child respond to his/her name when you call? | ○ | ○ | ○ | ○ |
| 15. If you point at a toy across the room, does your child look at it? | ○ | ○ | ○ | ○ |
| 16. Does your child walk? | Yes→ | ○ | ○ | ←No |
| 17. Does your child look at things you are looking at? | ○ | ○ | ○ | ○ |
| 18. Does your child make unusual finger movements near his/her face? | ○ | ○ | ○ | ○ |
| 19. Does your child try to attract your attention to his/her own activity? | ○ | ○ | ○ | ○ |
| 20. Have you ever wondered if your child is deaf? | ○ | ○ | ○ | ○ |
| 21. Does your child understand what people say? | ○ | ○ | ○ | ○ |
| 22. Does your child sometimes stare at nothing or wander with no purpose? | ○ | ○ | ○ | ○ |
| 23. Does your child look at your face to check your reaction when faced with something unfamiliar? | ○ | ○ | ○ | ○ |
| Section B: Interview (for investigator use only) | | | | |
| 1. During the appointment, has the child made eye contact with you? | Never  ○ | Rarely  ○ | Sometimes  ○ | Often  ○ |
| 2. Get child’s attention, then point across the room at an  interesting object and say “Oh look! There’s a [name of toy]!” Watch child’s face. Does the child look across to see what you are pointing at? |  |  | Yes  ○ | No  ○ |
| 3. Get the child’s attention, then give child a miniature toy cup and teapot and say, “Can you make a cup of tea?” Does the child pretend to pour out tea, drink it, etc.? |  | Yes  ○ | Imitate  ○ | Never  ○ |
| 4. Say to the child “Where’s the light?” or “Show me the light.” Does the child point with his/her index finger at the light? | Never  ○ | Point only  ○ | Look only  ○ | Point & look  ○ |
| 5. Can the child build a tower of bricks? (If so, how many?) (Number of bricks:_____) |  |  | Yes  ○ | No  ○ |

Core questions in section A includes question 2,5,7,9,13,15,23

*Scoring system*

Section A

In items 11, 18, 20 and 22, choice of “yes” (answers of sometimes or often) was regarded as positive. For items 16, no was considered as positive and yes was considered as positive.

In the remaining items, however, the choice of “no” (answers of never or rarely) was regarded as positive.

And then the “positive” was recorded as 1 point and the “negative” was recorded as 0 point. The total score ≥ 6 and the score of core items ≥ 2 were classified as having autism-like behaviors.

Section B

Item 1:It was defined positive as follows: for eye contact, never or seldom was considered positive, whereas usually or often was considered negative.

Item 2: For gaze monitoring, yes was considered negative and no was considered positive.

Item 3: For pretend play, negative was considered only when the child could play without imitation; imitation after demonstration by the interviewer or no pretend play was considered positive.

Item 4: For protodeclarative pointing, a pass (negative) was warranted only when the child could point and look at the object. All other responses (look only, point only, or never) were regarded as positive.

Item 5 (block building) was a test of the mental ability of the subject and was not used in the statistical tests.

And then the “positive” was recorded as 1 point and the “negative” was recorded as 0 point.

The total score ≥ 2 were classified as having autism-like behaviors for Section B.
